# Dietary Predictors and Plasma Concentrations of Perfluorinated Compounds in a Coastal Population from Northern Norway

**DOI:** 10.1155/2009/268219

**Published:** 2010-01-06

**Authors:** Charlotta Rylander, Magritt Brustad, Helena Falk, Torkjel M. Sandanger

**Affiliations:** ^1^Department of Community Medicine, University of Tromsø, 9037 Tromsø, Norway; ^2^Polar Environmental Centre, Norwegian Institute for Air Research, 9296 Tromsø, Norway

## Abstract

Dietary intake, age, gender, and body mass index were investigated as possible predictors of perfluorinated compounds in a study population from northern Norway (44 women and 16 men). In addition to donating a blood sample, the participants answered a detailed questionnaire about diet and lifestyle. Perfluorooctane sulfonate (PFOS) (29 ng/mL), perfluorooctanoate (PFOA) (3.9 ng/mL), perfluorohexane sulfonate (PFHxS) (0.5 ng/mL), perfluorononanoate (PFNA) (0.8 ng/mL), and perfluoroheptane sulfonate (PFHpS) (1.1 ng/mL) were detected in more than 95% of all samples. Of the dietary items investigated, fruit and vegetables significantly reduced the concentrations of PFOS and PFHpS, whereas fatty fish to a smaller extent significantly increased the levels of the same compounds. Men had significantly higher concentrations of PFOS, PFOA, PFHxS, and PFHpS than women. There were significant differences in PFOS isomer pattern between genders, with women having the largest proportion of linear PFOS. PFOS, PFHxS, and PFHpS concentrations also increased with age.

## 1. Introduction

As a result of many years of production and use in industry and consumer products, perfluorinated compounds (PFCs) are frequently found in the environment as well as in human blood world wide [1–4]. Due to their unique properties to repel both water and oil, PFCs have been important components of surface protectants for different materials and in fire-fighting foams and chemicals [5]. The predominant PFC in human samples is perfluorooctane sulfonate (PFOS), although other frequently detected compounds are perfluorooctanoate (PFOA), perfluorohexane sulfonate (PFHxS), and perfluorononanoate (PFNA) [1, 3]. In Norway, PFOS has mainly been used as a component in fire-fighting foams on oil rigs, although there is no information available about the use of PFHxS or PFOA [6]. Perfluorocarboxylates (PFCAs), for example, PFOA, and perfluoroalkyl sulfonates (PFASs), for example, PFOS, exist as branched and linear isomers. The linear isomer of PFOS is most common in technical mixtures and also in human samples [7, 8]. Differences in proportion of linear PFOS levels between inhabitants of three different countries have been reported and the authors concluded that this indicated different exposure sources [7]. However, there is limited information on isomer distribution in humans world wide. 

PFOS and PFHxS have been reported to bioaccumulate and magnify in the food chain [9, 10], even though PFCs behave differently from the legacy persistent organic pollutants (POPs) in the environment. A number of toxic effects, including alterations in fatty acid metabolism, enlargement of the liver, reduced birth weight, altered growth and development, and increased mortality in newborns, have been demonstrated in PFOS- and PFOA-exposed rodents [11–14]. Epidemiological studies on humans and health effects of PFCs are so far limited. Occupationally exposed workers in PFC industries have been studied in relation to morbidity, bladder cancer, and self-reported medical conditions [15–17]. No association between exposure and outcome was found. Several PFCs are found in human cord blood, indicating that these compounds easily cross the placental barrier. Results from human studies investigating the association between birth weight and PFC concentration are so far conflicting [18, 19]. In 2001, 3 M, one of the major producers of PFOS, voluntarily started to withdraw PFOS from production due to its persistent and toxic properties in the environment [20]. By 2015 or earlier, DuPont, which is a large producer of PFOA, is committed to totally phasing out PFOA and PFOA production precursors [21].

Even though PFCs have recently received a great deal of attention, routes of human exposure to PFCs are still unclear. Different pathways have been considered, such as diet, contaminated drinking water, household dust, and outdoor and indoor air [22]. Some studies have found diet to be the major pathway, whereas others suggest that diet is part of a more complex exposure scenario [22–25]. Five different studies have investigated PFC concentrations in food samples from Germany, Canada, the UK, Spain, and the Netherlands, and the results deviate [23–27]. None of the studies detected PFCs in all samples analyzed and several of them investigated only selected foodstuffs, not the whole diet. The UK food survey found the highest concentrations of PFOS in potatoes and potato products, such as french fries, hash browns, and potato salads [26]. The Spanish study [25] reported the highest concentrations of PFOS in fish and dairy products whereas the Canadian study [23] found the highest concentration of PFOS in beefsteak and salt water fish. De Voogt et al. reported the highest PFOS concentrations in beef, cod, and milk [27]. A study on seafood from Chinese fish markets observed detectable but low concentrations of PFOS (0.33–13.9 ng/g) in all species investigated, with the highest level in mantis shrimp [28]. Recently, the National Institute of Nutrition and Seafood Research in Norway reported low levels of PFCs in capelin (2–3.5 ng/g) and shrimp (<1–10 ng/g) and levels below the limit of detection (LOD) (<3 ng/g) in filets of farmed salmon [29, 30]. Only a few previous studies have investigated the relationship between self-reported dietary intake and plasma concentrations of PFCs [31–33]. Two of the studies [31, 33] concluded that locally caught fish significantly increased the body burden of PFOS. The third study [32] observed a positive association between PFOS and PFOA and consumption of red meat, snacks, and animal fat, and a negative relationship to intake of fruit, vegetables, and poultry.

The aim of this study was to determine the background concentrations of PFCs in a Norwegian coastal population in relation to age, gender, body mass index (BMI), and dietary habits, with special emphasis on fish consumption. This study also adds more information about the PFOS isomer pattern in human blood samples.

## 2. Materials and Methods

### 2.1. Study Participants

Study participants were recruited by an advertisement in the local newspaper through the survey “UV-light in Northern Norway and D-vitamin production in skin”, a research project on Andøya Island, described elsewhere [34]. The people on Andøya are known to have a high consumption of various kinds of seafood. Criteria for being included in the study were age between 20 and 60 years and living in the municipality of Andenes at 69°N. The participants were 44 women and 16 men of various ages (26–60 years) (Table 1). Concentrations of POPs in the same study group have been reported elsewhere [35].

### 2.2. Food Frequency Questionnaire

The participants answered the Norwegian Women and Cancer Study's (NOWAC) food frequency questionnaire. Study participants were asked to record how often they consumed 86 different foodstuffs including alcohol. Questions about portion size were also answered. The questionnaire differentiates between different kinds of fish and meat consumed. These variables were later grouped together (Table 1). The questionnaire has been validated and described in detail elsewhere [36, 37]. 

### 2.3. Blood Samples

Blood samples were collected in August to September 2005. Blood was drawn in BD Vacutainer blood collection tubes (BD, NJ, USA) containing EDTA buffer (10.8 mg) and mailed overnight to the University of Tromsø. The samples were centrifuged at 3000 rev./min for 15 min and the plasma was collected. Plasma samples were stored at −80°C until analysis.

### 2.4. Chemical Analysis

Samples were extracted and cleaned up using a modified method from Powley et al. [38]. In short, 0.2 g plasma was weighed into a 50 mL polypropylene centrifuge tube (Nalgene, Rochester, NY, USA). Twenty-five microliters of ^13^C_4_-labeled PFOS and ^13^C_4_-labeled PFOA (0.1 ng/*μ*L) (Wellington laboratories, Guelph, ON, Canada) and 4 g methanol (Merck, Darmstadt, Germany) were added to the test tube as internal standards and extraction solvents. Samples were extracted for 3 × 10 minutes in an ultrasonic bath (Branson Ultrasonics BV, Soest, the Netherlands). In between each extraction, samples were mixed thoroughly with a vortex mixer (VWR, Wester Chester, PN, USA). Samples were then centrifuged at 2000 rev./min for 5 min using a Jouan A14 centrifuge (Thermo Scientific, Waltham, MA, USA). The supernatant was collected before volume reduction to 1 g on a Rapid Vap evaporation unit (Labconco, Kansas City, MO, USA). The extract was transferred to a 1.5 mL microcentrifuge tube (Brand GMBH, Wertheim, Germany) containing 25 mg ENVI-Carb 120/400 (Supelco, PN, USA) and 50 *μ*L glacial acetic acid (KEBOLab, Kalbakken, Norway). The solution was mixed thoroughly and then centrifuged for 10 min at 10 000 rev./min. The supernatant was weighed and transferred to a 1.8 mL glass tube, and 20 *μ*L 3,5-bis(trifluoromethyl)phenyl acetic acid (BTPA) (0.1 ng/*μ*L) (Wellington Laboratories, Guelph, ON, Canada) were added as recovery standard. Before analysis, 100 *μ*L extract was mixed with 100 *μ*L water containing 2 mmol/L NH_4_OAc (BDH Laboratory Supplies, Leicestershire, England).

PFCs were analyzed using a quadrupole time-of-flight- mass spectrometer, Q-TOF micro, equipped with a 2777 autosampler and a binary HPLC pump (1525) from Waters (Milford, MA, USA). The method used is slightly modified from that of Berger and Haukås [39]. Mobile phases consisted of (a) 2 mmol/L NH_4_OAc in water and (b) 2 mmol/L NH_4_OAc in methanol (Merck, Darmstadt, Germany). Before analysis, mobile phases were degassed using an ultrasonic bath (Branson Ultrasonics BV, Soest, the Netherlands). A 50 *μ*L sample was injected into an ACE 3 C18 reversed-phase column, with particle size 3 *μ*m and length 150 mm (ACT, Aberdeen, Scotland). The flow rate of the mobile phase was 0.2 mL/min. The following gradient settings were applied for elution of the target analytes from the column: 0 min to 50% B; 0–5 min linear gradient to 85% B; 5–10 min to 85% B; 10-11 min linear gradient to 99% B; 11–20 min to 99% B; 20-21 min linear gradient to 50% B; 21–28 min to 50% B. The QTOF-MS was operated by the Mass Lynx 4.1 software in negative electrospray ionization mode (ESI) in the *m/z* range 100–725. Settings were optimized before analysis as follows: capillary voltage, −3 kV; sample cone voltage, 50 V (0.5–10.3 min), 35 V (0.5–20.0 min), and 20 V (10.3–20.0 min); desolvation and source temperature, 350 and 120°C, respectively; nitrogen was used as cone gas at a flow of 20 l/min, as nebulizer gas maximum flow, and as desolvation gas at 600 l/min. Target analytes and analytical standards, their abbreviation, quantification masses and cone voltages are listed in supplementary Table S1 in Supplementary Material available online at doi:10.1155/2009/268219. 

The quantification was conducted by the QuanLynx software, version 3.5 (Waters, Milford, MA, USA).

The linear PFOS isomer was chromatographically separated from the branched isomers and quantified, both separated and as the sum of all isomers. The coelution of branched isomers (one peak) was not structurally elucidated but rather identified as eluting earlier than the linear PFOS, as indicated in supplementary Figure S1. Isomer specification was not performed for the other PFCs, where the linear isomer clearly dominated. Data presented as “PFOS” consist of the sum of the linear and the coeluted peak of  branched isomers. Similar response factors have been reported for branched and linear isomers of PFOS [40], so the mass labeled “internal standard for linear PFOS” was used for quantification of the branched isomer as well.

### 2.5. Quality Control of Chemical Analysis

The quality of the analysis was assured through repetitive analysis of blank samples and reference samples obtained from previous international comparison programs. For each batch of 30 samples, one reference material and two blank samples were prepared. Three times a year, the present laboratory also participates in the AMAP Ringtest for Persistent Organic Pollutants in Human Serum, an international comparison program, organized by Institut National de Santé Publique du Québec, Canada. Results from interlaboratory comparisons indicate that the uncertainties of our analysis are well within ±30% of the assigned values. Four samples were excluded from all analysis due to poor chromatography. Therefore, the total number of study participants was reduced to 56. Recovery rates varied between 60% and 120%.

The LOD was automatically calculated by the quantification software from the signal-to-noise level in each sample. The individual LODs were comparable for all samples and an average is reported for each analyte in Table 2. PFOA was detected in a few blank samples. If the concentration of PFOA in these samples was larger than the software-determined LOD for that batch of samples, LOD was determined from the concentration of PFOA in the blanks. All samples were well within the linear range of the instrument.

### 2.6. Statistical Analysis

Statistical analysis was performed using the freely available software R, version 2.8.1 (http://www.cran.r-project.org/). Statistics for PFOSA, which had more than 20% of the observations below LOD, were performed with the NADA package for R. Summary statistics for PFOSA were calculated using the maximum likelihood estimation (MLE) according to Helsel [41]. PFHpA had more than 95% of the observations below LOD and were not evaluated statistically.

Possible predictors of PFC concentrations investigated were age, gender, BMI, and nine different categories of foodstuff (see Table 1). The impact of these predictors on PFOS, PFOA, PFHxS, PFHpS, PFNA, and percentage linear PFOS was investigated using linear models on log-transformed variables or Wilcoxon's rank sum test. For the censored data (PFOSA), the nonparametric Peto-Prentice test, as well as the nonparametric Akritas-Thiel-Sen slope in the NADA package for R, was used. The censored methods are described by Helsel [41]. Model assumptions for the linear models were evaluated using diagnostic plots of the residuals. Parameter estimates (*β*) with 95% confidence interval and the levels of significance (*P* values) for the final regression models are reported in Table 3. The parameter estimates (*β*) are back-transformed logresults and should be interpreted as the number of times that the response variable increased/decreased by one unit in explanatory variable. Wilcoxon's test estimator (*W*) and the corresponding *P* values are reported in the text. The *P* values <.05 were considered to be significant.

## 3. Results

Median, arithmetic mean, range, LOD, and percentage of samples with values> LOD of the eight monitored PFCs are provided in Table 2. PFOS, PFOA, PFHxS, PFHpS, and PFNA were detected in more than 95% of all samples.

**Figure 1:**
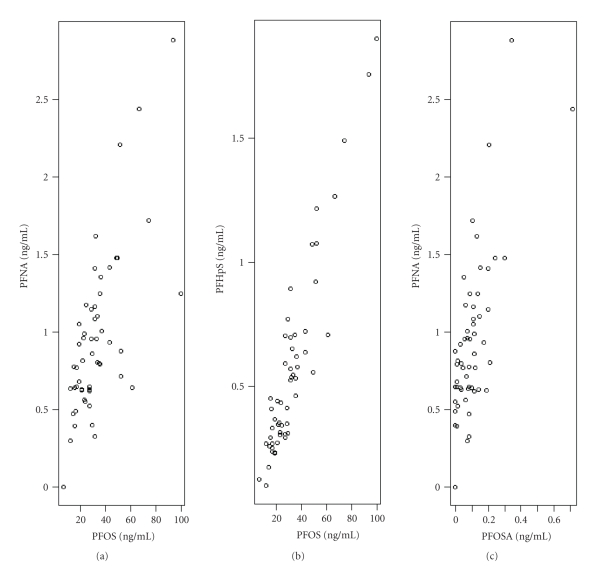
Correlation between perfluorooctane sulfonate (PFOS) and perfluorononanoate (PFNA) (*r* = 0.70) and PFOS and perfluoroheptane sulfonate (PFHpS) (*r* = 0.93), as well as perfluorooctane sulfonic acid (PFOSA) and PFNA (*r* = 0.72).

PFOS and PFHpS concentrations were strongly correlated (*r* = 0.93) (Figure 1), as well as PFOS and PFNA (*r* = 0.70) and PFOSA and PFNA (*r* = 0.72) (Figure 1). The remaining PFCs were medium strong or weakly correlated (supplementary Table S2). There were four significant predictors for PFOS concentration in this study group: gender, age, intake of fruit and vegetables, and intake of fatty fish (Table 3). Men had 75% higher concentrations of PFOS than those of women and the PFOS concentration increased by 2% per year of age (for the whole group) (Table 3). An additional serving (150 g) of fruit and vegetables per day during the last year decreased the concentrations of PFOS by 16%, whereas an extra meal (150 g) of fatty fish per week during the last year resulted in an increase in PFOS concentrations (22%) (Table 3). Age, gender, consumption of fatty fish, and consumption of fruit and vegetables explained 57% of the variation in PFOS concentration (Table 3). Fatty fish alone accounted for 4%, fruit and vegetables explained 16%, and age and gender 37%. There was no correlation between intake of fatty fish and intake of fruit and vegetables (*r* = −0.00014). 

Male sex was clearly associated with increased plasma concentrations of PFOA (44%), PFHxS (172%), and PFHpS (107%) (Table 3). There was a positive relationship between age and PFHxS and PFHpS, both indicating a 3% increase in concentrations per year of age (Table 3). An additional intake of 150 g (one serving) of fatty fish per week, over the last year, resulted in increased concentrations of PFHpS (32%), whereas an additional serving (150 g) of fruit and vegetables each day, over the last year, decreased the concentration of PFHpS by 17% (Table 3). None of the investigated variables influenced the concentration of PFNA or PFOSA significantly. Two people were excluded from the PFOS and PFHpS models because they were strongly influential. The results were the same, before and after exclusion, but the refined models explained more of the variation in the dataset. Intake frequencies of fatty fish and fruit/vegetables, and the corresponding concentrations of PFOS and PFHpS, are reported in Table S3. The person who consumed two to three servings of fatty fish per week (67 ng/mL PFOS, supplementary Table S3) was not indicated as an outlier by the diagnostic plots of the residuals, although, on removal of that person from the dataset, fatty fish were no longer a significant predictor (*P* > .05) for PFOS or PFHpS concentrations. The result for fruit and vegetables, age, and gender remained the same, before and after exclusion. In the final model (Table 3), the person was, however, included because of the indication of not being an outlier.

The study group had an average proportion of linear PFOS of 69% (range 49%–100%) (Table 2). Women had a significantly larger percentage of linear PFOS than that of men (70% versus 67%, *W* = 415.5, *P* = .047). The proportion of linear PFOS varied between 49% and 100% for men and between 56% and 100% for women (Table 2).

## 4. Discussion

We have shown that PFOS and PFHpS concentrations in the current study decreased with intake of fruit and vegetables and increased with intake of fatty fish. An additional serving of fruit and vegetables (150 g) per day, over the last year, gave an estimated decreased body burden of PFOS and PFHpS of 16% (95% confidence interval (CI): 8%–25%) and 17% ((CI): 6%–29%), respectively. On the other hand, an additional meal of fatty fish per week (150 g), over the last year, resulted in 22% (CI: 2%–45%) and 32% (CI: 3%–68%) higher concentrations of PFOS and PFHpS, respectively. Intake of fatty fish and intake of fruit and vegetables were not correlated and the observed effect cannot be explained by co-variation. The maximum intake of fruit and vegetables was 6.6 servings per day and 2.3 servings of fatty fish per week (supplementary Table S3). No conclusions outside these intake ranges can be made from this dataset.

Surprisingly, consumption of fruit and vegetables decreased PFOS concentrations in the current study. Supporting and contradictory findings have been reported by others. Halldorsson et al. [32] found that intake of fruit and vegetables decreased PFOS and PFOA concentrations in the Danish Birth cohort (*n* = 1076), but the effect could be partly explained by lower intake of red meat, animal fats, and snacks (positively associated to PFOS) among the high consumers of fruit and vegetables. Emmet et al. [42] reported a positive association between locally grown vegetables and PFOA concentration, whereas Holzer et al. [31] observed no effect from intake of locally grown fruit and vegetables on PFOA, PFOS, or PFHxS concentrations in a group of 521 participants. The different findings may reflect different study designs and/or presence of confounding factors. In the Norwegian Women and Cancer Study (NOWAC), dietary patterns and lifestyle factors were investigated in 35 554 Norwegian women [43]. “Healthy eaters” had the highest intake of fruit, vegetables, skimmed milk, juice, instant coffee, crisp bread, rice, chicken, and cod liver oil, which is frequently used as a food supplement in Norway. They were also characterized by high education, high income, few current smok, and high activity. They were more likely to use dietary supplements than the other women and most of them lived in the southern or eastern part of Norway. This study indicates that health awareness is characterized not only by diet but also by many lifestyle factors. Consequently, our findings of decreasing PFOS and PFHpS concentrations with increasing intake of fruit and vegetables may be explained by a large number of confounding variables that characterize the lifestyle of “healthy eaters”, rather than a high fruit and vegetable intake. It is nevertheless an important finding that needs to be investigated further.

In the current study, 20% of the observed variation in PFOS concentration were explained by intake of fruit and vegetables (16%) and intake of fatty fish (4%). These findings support the previous hypothesis that fish intake contributes to increased body burdens of PFOS [31, 33]. However, only one person (67 ng/mL, supplementary Table S3) was responsible for the significant association of fatty fish (*P* < .05), and interpretation of this result should therefore be made with care. In addition, of the participants' total intake of seafood, only 9% consisted of fatty fish. The remaining 91% were intake of lean fish, fish products, and other kinds of fish that had no impact on PFC concentrations. Thus, the main source of seafood did not contribute to increased body burdens of PFOS or PFHpS. The health effects of seafood consumption have been frequently debated. Benefits from a fish-rich diet, for example, lower risk of cardiovascular diseases, has been weighed up against the possible drawbacks of increased body burden of environmental pollutants [44]. A substantial increase of one meal of fatty fish per week over a year resulted in only a 22% increase in PFOS concentration, which corresponds to 6 ng/mL (from 29 ng/mL (median) to 35 ng/mL). Furthermore, no associations between a number of outcomes and occupational exposure to PFCs have been found in a study group exposed to higher concentrations than the general population [15–17]. It is therefore unlikely to expect adverse effects from intake of fatty fish. The protective effects of seafood on several health outcomes are clear and well documented [45] and the benefits of a fish-rich diet should by far outweigh the concerns. PFCs are, in addition, detected in all kinds of food [23, 25, 26, 28] and also in household dust [46], drinking water [42], and consumer products [47], indicating that the exposure is complex and could not be explained just by single foodstuffs. This was also recently pointed out by Halldorsson et al. [32] that suggested that the overall diet is likely to be more important for PFC exposure than single foodstuffs and results from individual food groups should therefore be interpreted with care. Kärrman et al. [48] concluded also that the importance of dietary intake of PFCs may differ between regions. 

Fatty fish and fruit/vegetables were also significant predictors for PFHpS concentrations in this study group. This finding is most probably an artifact, explained by the strong correlation between PFOS and PFHpS (*r* = 0.93). However, there was a lack of association between the investigated dietary predictors and plasma concentrations of PFOA, PFHxS, PFNA, and PFOSA in this study group. This may point to the diet being a less important pathway for these compounds due to low concentrations present in food. The UK food survey detected PFOS in some of their composite food samples, whereas the concentrations of PFOA were below LOD (<0.5–10 ng/g) in the same samples [26]. Ericson et al. [25] and Tittlemier et al. [23] detected PFOS in nearly all of their composite food samples, although only a few had detectable concentrations of PFOA and/or PFHpA and PFNA. PFOSA was not found in any of these food surveys. Another explanation for the lack of association between the dietary variables and PFOA, PFHxS, PFNA, and PFOSA could be the fact that human blood concentrations of these compounds are lower and more uniform. Also, the current study had fewer participants, that is, lower chance of detecting weak effects.

All the investigated PFCs (except for PFOSA versus PFOA and PFHxS) were medium strong or highly correlated (supplementary Table S2), with the strongest correlation being between PFOS and PFHpS (*r* = 0.93). The correlation between PFCs seems to vary between countries/studies. Olsen et al. [49] reported the strongest correlation between PFOS and PFOA (*r* = 0.63) in American Red Cross blood donors. Similar results were provided by Haug et al. [50] that reported PFOS and PFOA being highly correlated (*r* = 0.95) in pooled Norwegian samples. Ericson et al. [51] observed the strongest correlation between PFOS and PFHxS (*r* = 0.23) in a study group from Spain while Rylander et al. [52] reported the strongest correlation between PFOS and PFHxS (*r* = 0.94) in delivering women from Vietnam. Differences may be attributable to a number of factors, including different sample sizes, age and gender compositions of the study groups, different exposure sources, or analytical challenges.

Men had significantly higher concentrations of PFOS, PFOA, PFHxS, and PFHpS than those of women in this study group. Several other studies have reported gender-related differences for the same compounds, with men having higher concentrations than those of women [31, 51, 53–55]. PFOS and PFOA have been found to cross the placental barrier and have been detected in human breast milk, in addition to PFHxS, PFOSA, and PFNA [18, 19, 56–60]. Differences between genders may therefore be partly explained by transplacental transfer and excretion through breastfeeding, which seem likely in this rather young study group. The differences attributable to childbearing are expected to decrease over time due to continuous exposure to contaminants. Another possible explanation for the gender-related differences could be a higher dietary intake among men. Menopause status, use of contraceptives, and blood donor practices among the study participants could also affect the results through the possible blood loss, but this was not taken into account in the current study. These variables have, however, not been identified as confounding factors in other studies.

Despite the fact that men had higher concentrations of PFOS than those of women in the current study, female blood contained a significantly larger proportion of linear PFOS compared with male blood (70% versus 67%). The major production process of PFOS, electrochemical fluorination (ECF), produced 70%–80% linear PFOS [8]. Our samples contained on average 69% linear PFOS (range 49%–100%). This is comparable to results from Sweden (68%, range 50%–70%) [7] and a previous study in Norway (50%–78%) [50], but higher than in samples from Australia (59%) [7] and the UK (60%) [7] and considerably lower than in samples from Vietnam (83%) [52]. Powley et al. [38] showed recently that the proportion of branched PFOS differed between Arctic cod and ringed seal (which feed on Arctic cod), indicating differences in elimination rates between species. Thus, the dietary contributions of PFOS isomers may vary between countries/continents due to different dietary habits. Also, the importance of different exposure routes, for example, diet, dust, and consumer products, could vary between countries. In the current study, the branched isomers of PFOS were not chromatographically separated but quantified as the sum of a mixture of branched isomers. Kärrman et al. [7] as well as Benskin et al. [61] identified several of the branched isomers of PFOS in human blood, and both studies indicate that the monoperfluoromethyl-substituted isomers are most abundant in human blood samples. However, in order to make correct comparisons of PFOS distribution world wide, it is important to specify whether linear PFOS or the sum of all PFOS isomers is reported, especially as the branched isomers may contribute as much as 30% to the total.

PFOS concentrations in this study group were comparable to samples from Denmark, Sweden, Australia, the USA, Canada, Poland, Korea, and Belgium, although samples from Spain, Germany, Colombia, Brazil, Italy, India, Sri Lanka, Vietnam, and Malaysia were on average up to 20 times less contaminated [3, 31, 51–53, 62, 63]. Poland, Korea, and Japan have reported high PFOA concentrations, whereas studies from China and Spain reported low levels [2, 51, 54]. Our samples were in the midrange. There were smaller differences in PFHxS concentrations between countries and only a few studies reported the concentrations of the other PFCs. Vietnamese women, for example, had considerable lower concentrations of PFHpS but comparable levels of PFNA to the current study group [52]. Considerable regional differences in PFC concentrations within countries [48, 54] have also been observed, indicating that a large population-based study is needed to achieve the full picture of the exposure pattern within a country.

We observed a positive relationship between age and PFOS, PFHxS, and PFHpS concentrations. Previous studies show contradictory results regarding age-related differences in PFC concentrations [2, 31, 53], which point to no clear age trend for the PFCs.

## 5. Conclusions

Intake of fruit/vegetables and fatty fish affected the concentration of PFOS and PFHpS in this study group. The reason for the decreased PFOS and PFHpS concentrations with increased intake of fruit and vegetables has yet to be explained. A larger population based study is suggested to evaluate the effect of fatty fish consumption on PFOS/PFHpS concentrations thoroughly since uncertainties were present in the current study (only one person responsible for the significant effect). Dietary predictors should, however, be interpreted with care since dietary habits also reflect differences in lifestyle (not adjusted for in the current analysis) which may affect PFC concentrations. Future research should focus on dietary patterns instead of single food groups and lifestyle factors should also be taken into account. Men had significantly higher concentrations of PFOS, PFOA, PFHxS, and PFHpS than those of women, and PFOS, PFHxS, and PFHpS increased with age. Women had a larger proportion of linear PFOS than those of men (70% versus 67%). However, the overall average of linear PFOS in this study group was comparably to previous studies from Sweden and Norway but considerable higher than in samples from Vietnam. In future studies the PFOS isomers should be reported separately in order to help identify differences between populations and different sources of exposure. 

## Supplementary Material

The supplementary material contains information about target analytes, standards and instrumental settings, as well as a chromatogram showing branched and linear PFOS. The correlation coefficients for the investigated PFCs and intake frequencies of fatty fish and fruit and vegetables and corresponding PFOS and PFHpS concentrations are also provided in the supplementary material.

## Figures and Tables

**Table 1:** Study group characteristics.

	Total *n* = 56^a^ (100%)	Women *n* = 41 (73%)	Men *n* = 15 (27%)
	Median (range)	Median (range)	Median (range)

Age (years)	44 (26–60)	44 (26–60)	44 (32–57)
BMI (kg/m^2^)	25 (18–43)	24 (18–43)	25 (21–33)
Intake of meat (kg/year)^b^	46 (5.2–119)	42 (5.2–119)	63 (12–83)
Intake of dairy products (kg/year)	65 (40–299)	54 (4.0–299)	138 (8.9–235)
Intake of bread and cereals (kg/year)	48 (14–114)	46 (14–108)	78 (21–114)
Intake of vegetables and fruit (kg/year)^c^	151 (51–363)	154 (52–363)	136 (51–305)
Intake of fatty fish (kg/year)^d^	3.2 (0–39)	3.1 (0–18)	3.4 (0–39)
Intake of lean fish (kg/year)^e^	15 (0–63)	14 (0–63)	18 (5.1–38)
Intake of other kinds of fish (kg/year)^f^	2.1 (0–19)	2.1 (0–19)	2.1 (0–19)
Intake of fish products (kg/year)^g^	15 (1.7–53)	15 (1.7–41)	20 (4.5–53)
Intake of shellfish (kg/year)	0 (0–3.2)	0 (0–3.2)	0 (0–3.2)

^a^Four people were excluded due to poor chromatography and the total number of study participants was reduced to 56.

^b^Include meat and meat products.

^c^Include potatoes, vegetables, juice, jam, and fruit.

^d^Include intake of salmon, mackerel, wolfish, and herring.

^e^Include intake of boiled and fried cod.

^f^Include intake of other kinds of fish not included in above two categories.

^g^Include intake of fish cakes, fish au gratin, deep-fried fish, and fish spread.

**Table 2:** PFC concentrations (ng/mL) in the study group.

Concentration (ng/mL plasma)	Total *n* = 56					Men *n* = 15			Women *n* = 41		
	Median	AM	Range	LOD	% >LOD	Median	AM	Range	Median	AM	Range
PFOSA	0.08	0.11	<LOD–0.7	0.03	79	0.11	0.11	<LOD–0.35	0.08	0.10	<LOD–0.71
PFHxS	1.1	1.8	0.40–13	0.07	100	1.8	3.5	0.95–13	0.8	1.2	0.40–3.8
PFHpS	0.46	0.57	<LOD–1.9	0.13	96	0.70	0.89	0.31–1.9	0.35	0.45	<LOD–1.3
PFOS branched	9.4	10	<LOD–31	0.22	96	12	15	<LOD–31	7.1	8.3	<LOD–26
PFOS linear	20	23	4.7–69	0.40	100	24	33	14–69	17	19	4.7–47
PFOS	29	33	6.9–99			43	48	28–99	24	27	6.9–67
PFHpA	NA	NA	NA	0.26	0	NA	NA	NA	NA	NA	NA
PFOA	3.9	4.4	1.4–9.6	0.30	100	5.1	5.4	3.0–8.8	3.4	4.0	1.4–9.6
PFNA	0.81	0.95	<LOD–2.9	0.26	98	0.94	1.1	0.40–2.9	0.77	0.88	<LOD–2.4
% linear PFOS	69	69	49–100			67	67	49–100	69	70	56–100

AM, arithmetic mean; LOD, method detection limit; % >LOD, percentage of samples in which the analyte was detected; NA, not available; PFOSA, perfluorooctane sulfonic acid; PFHxS, perfluorohexane sulfonate; PFHpS, perfluoroheptane sulfonate; PFOS, sum of branched and linear isomers of PFOS (perfluorooctane sulfonate); PFHpA, perfluoroheptanoate; PFOA, perfluorooctanoate; PFNA, perfluorononanoate; % linear PFOS, percentage linear PFOS related to PFOS.

**Table 3:** Back-transformed parameter estimates, 95% confidence interval (CI) and *P* values for the final regression models of selected PFCs and significant predictors.

	PFOS		PFOA		PFHxS		PFHpS	
	*β* (95% CI)	*P*	*β* (95% CI)	*P*	*β* (95% CI)	*P*	*β* (95% CI)	*P*
Male sex	1.75 (1.43, 2.14)	<.001	1.44 (1.12, 1.85)	.009	2.72 (1.86, 3.92)	<.001	2.07 (1.57, 2.74)	<.001
Age (years)	1.02 (1.01, 1.03)	<.001	1.01 (0.998, 1.02)	.096	1.03 (1.01, 1.03)	<.001	1.03 (1.01, 1.04)	<.001
Consumption of fatty fish (150 g per week during a year)	1.22 (1.02, 1.45)	.029					1.32 (1.03, 1.68)	.027
Consumption of fruit and vegetables (150 g per day during a year)	−1.16 (−1.08, −1.25)	<.001					−1.17 (−1.06, −1.29)	.002
*R* ^2^ (%)	57		17		41		50	

*β*, parameter estimates (back-transformed logresults); *R* ^2^, coefficient of determination, that is, the proportion of variability in the dataset that is explained by the model; PFOS, perfluorooctane sulfonate; PFOA, perfluorooctanoate; PFHxS, perfluorohexane sulfonate; PFHpS, perfluoroheptane sulfonate.
