# Supplementary material for: Dietary Predictors and Plasma Concentrations of Perfluorinated Compounds in a Coastal Population from Northern Norway
Source: J Environ Public Health. 2010 Jan 6;2009:268219. doi: 10.1155/2009/268219 (PMC2810450; doi:10.1155/2009/268219)
Supplement: Supplementary file 1 — The supplementary material contains information about target analytes, standards and instrumental settings, as well as a chromatogram showing branched and linear PFOS. The correlation coefficients for the investigated PFCs and intake frequencies of fatty fish and fruit and vegetables and corresponding PFOS and PFHpS concentrations are also provided in the supplementary material. [file 268219.f1.pdf]

Table S1. Target analytes and analytical standards, their abbreviation, quantification masses and cone voltages.

Compound	Abbreviation	Quantification mass (m/z) (Cone voltage (V))
3,5-Bis(trifluoromethyl)phenyl acetic acid[1]	BTPA	227 (35)
Mass labelled Perfluorooctanoate[2]	13 C-PFOA	372 (35)
Mass labelled Perfluorooctane sulfonate[2]	13C-PFOS	503 (50)
Perfluorooctanesulfonic acid	PFOSA	498 (35)
Perfluorohexane sulfonate	PFHxS	399 (50)
Perfluoroheptane sulfonate	PFHpS	449 (50)
Perfluorooctane sulfonate branched isomers	PFOS branched	499 (50)
Perfluorooctane sulfonate linear isomer	PFOS linear	499 (50)
Perfluoroheptanoate	PFHpA	319 (35)
Perfluorooctanoate	PFOA	369 (35)
Perfluorononanoate	PFNA	419 (35)

1.Used as recovery standard. 2. Used as internal standard. 3. Used as internal standard.

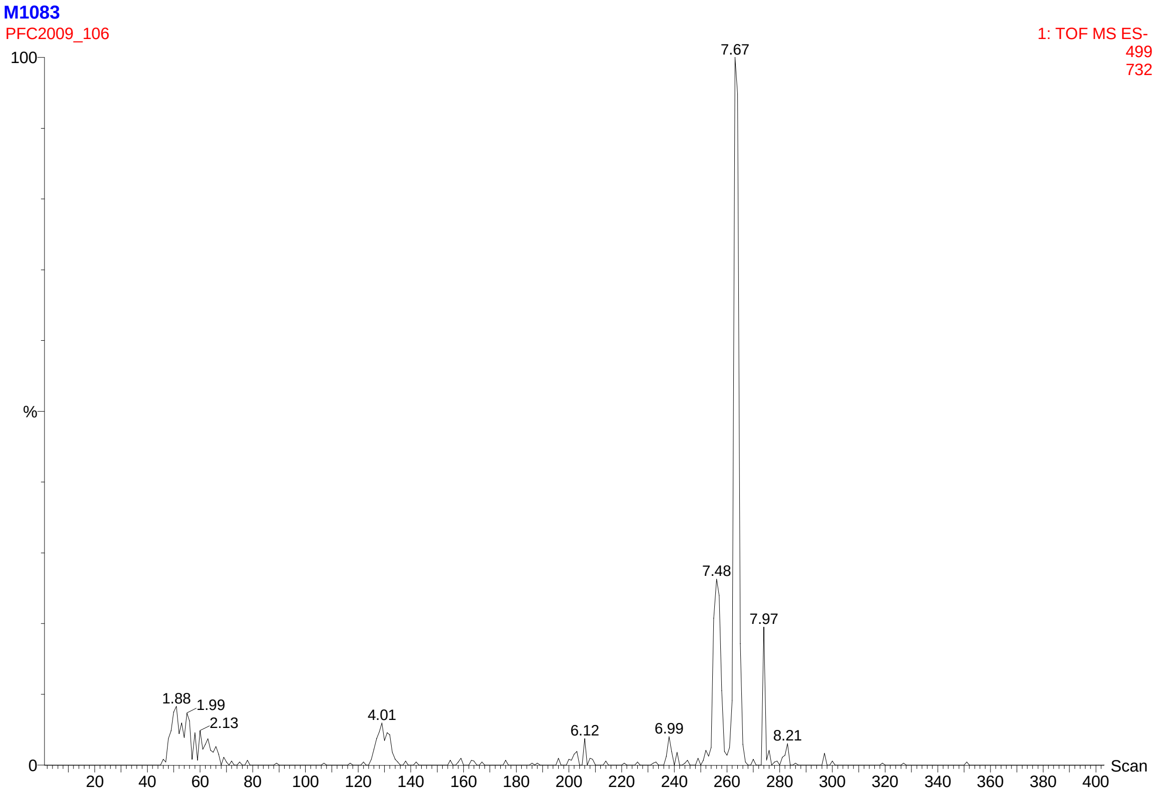

Fig S1. Chromatogram showing branched PFOS isomers at retention time 7.48 and the linear isomer at retention time 7.67.
